# Supplementary material for: Tonal and syllabic encoding in overt Cantonese Chinese speech production: An ERP study
Source: PLoS One. 2023 Dec 15;18(12):e0295240. doi: 10.1371/journal.pone.0295240 (PMC10723706; doi:10.1371/journal.pone.0295240)
Supplement: S1 Appendix — (DOCX) [file pone.0295240.s001.docx]

**Appendix**

List of targets and distractors

| Target | |  | Tonal Syllable related | | | Atonal Syllable related | | | Tone alone related | | |
| --- | --- | --- | --- | --- | --- | --- | --- | --- | --- | --- | --- |
| 飛機 | fei1 | gei1 | 非常 | fei1 | soeng4 | 肥胖 | fei4 | bun6 | 蝦餃 | haa1 | gaau2 |
| 糖果 | tong4 | gwo2 | 堂兄 | tong4 | hing1 | 躺下 | tong2 | haa6 | 存貨 | cyun4 | fo3 |
| 長褲 | coeng4 | fu3 | 牆壁 | coeng4 | bik1 | 搶奪 | coeng2 | dyut6 | 回收 | wui4 | sau1 |
| 荒島 | fong1 | dou2 | 方便 | fong1 | bin6 | 放棄 | fong3 | hei3 | 尖銳 | zim1 | jeoi6 |
| 汽車 | hei3 | ce1 | 戲劇 | hei3 | kek6 | 喜悅 | hei2 | jyut6 | 報紙 | bou3 | zi2 |
| 人腦 | jan4 | nou5 | 仁愛 | jan4 | ngoi3 | 忍受 | jan2 | sau6 | 嘗試 | soeng4 | si3 |
| 乳豬 | jyu5 | zyu1 | 語言 | jyu5 | jin4 | 迂腐 | jyu1 | fu6 | 倍數 | pui5 | sou3 |
| 斧頭 | fu2 | tau2 | 苦惱 | fu2 | nou5 | 富有 | fu3 | jau5 | 改造 | goi2 | zou6 |
| 樹木 | syu6 | muk6 | 豎起 | syu6 | hei2 | 暑假 | syu2 | gaa3 | 右腳 | yau6 | goek3 |
| 空樽 | hung1 | zeon1 | 兇殘 | hung1 | caan4 | 紅豆 | hung4 | dau2 | 分寸 | fan1 | cyun3 |
| 烏龜 | wu1 | gwai1 | 污染 | wu1 | jim5 | 狐狸 | wu4 | lei2 | 卑微 | bei1 | mei4 |
| 貝殼 | bui3 | hok6 | 輩份 | bui3 | fan6 | 背書 | bui3 | syu1 | 性質 | sing3 | zat1 |
| 皮鞋 | pei4 | haai4 | 脾胃 | pei4 | wai6 | 屁股 | pei3 | gu2 | 廚師 | cyu4 | si1 |
| 紙巾 | zi2 | gan1 | 子女 | zi2 | neoi5 | 治療 | zi6 | liu4 | 躲藏 | do2 | cong4 |
| 齒輪 | ci2 | leon4 | 恥辱 | ci2 | juk6 | 癡心 | ci1 | sam1 | 古蹟 | gu2 | zik1 |
| 公雞 | gung1 | gai1 | 功課 | gung1 | fo3 | 貢獻 | gung3 | hin3 | 得罪 | dak1 | zeoi6 |
| 蘋果 | ping4 | gwo2 | 平靜 | ping4 | zing6 | 拼音 | ping3 | jam1 | 巡視 | ceon4 | si6 |
| 風扇 | fung1 | sin3 | 蜂蜜 | fung1 | mat6 | 縫合 | fung4 | hap6 | 簽名 | cim1 | meng2 |
| 獅子 | si1 | zi2 | 思維 | si1 | wai4 | 試驗 | si3 | jim6 | 粗暴 | cou1 | bou6 |
| 青蛙 | cing1 | waa1 | 稱讚 | cing1 | zaan3 | 程序 | cing4 | zeoi6 | 觸犯 | zuk1 | faan6 |
| 磁鐵 | ci4 | tit3 | 持久 | ci4 | gau2 | 刺身 | ci3 | san1 | 符合 | fu4 | hap6 |
| 粟米 | suk1 | mai5 | 縮短 | suk1 | dyun2 | 熟練 | suk6 | lin6 | 僵持 | goeng1 | ci4 |
| 毛筆 | mou4 | bat1 | 無奈 | mou4 | noi6 | 務實 | mou6 | sat6 | 巢穴 | caau4 | jyut6 |
| 醫生 | ji1 | saang1 | 衣服 | ji1 | fuk6 | 倚靠 | ji2 | kaau3 | 初選 | co1 | syun2 |
| 兔仔 | tou3 | zai2 | 套餐 | tou3 | caan1 | 逃亡 | tou4 | mong4 | 對話 | deoi3 | waa6 |
| 城堡 | sing3 | bou2 | 繩索 | sing3 | sok3 | 聲音 | sing1 | jam1 | 頹廢 | teoi4 | fai3 |
| 手槍 | sau2 | coeng1 | 守護 | sau2 | wu6 | 壽命 | sau6 | ming6 | 採購 | coi2 | kau3 |
| 眉豆 | mei4 | dau2 | 微笑 | mei4 | siu3 | 美麗 | mei5 | lai6 | 浮躁 | fau4 | cou3 |
| 香蕉 | hoeng1 | ziu1 | 鄉下 | hoeng1 | haa2 | 享受 | hoeng2 | sau6 | 金幣 | gam1 | bai6 |
| 獸醫 | sau3 | ji1 | 瘦削 | sau3 | soek3 | 修改 | sau1 | goi2 | 記載 | gei3 | zoi3 |
| 樂譜 | ngok6 | pou2 | 愕然 | ngok6 | jin4 | 惡霸 | ngok3 | baa3 | 重要 | zung6 | jiu3 |
| 門口 | mun4 | hau2 | 瞞騙 | mun4 | pin3 | 滿意 | mun5 | ji3 | 營救 | jing4 | gau3 |
| 信件 | seon3 | gin2 | 迅速 | seon3 | cuk1 | 純真 | seon4 | zan1 | 霸道 | baa3 | dou6 |
| 旗幟 | kei4 | ci3 | 奇妙 | kei4 | miu6 | 企業 | kei5 | jip6 | 慚愧 | caam4 | kwai5 |
| 箭靶 | zin3 | baa2 | 戰爭 | zin3 | zang1 | 展覽 | zin2 | laam3 | 滲漏 | sam3 | lau6 |
| 神父 | san4 | fu6 | 晨光 | san4 | gwong1 | 辛酸 | san1 | syun1 | 潛水 | cim4 | seoi2 |
| 武器 | mou5 | hei3 | 舞蹈 | mou5 | dou6 | 帽子 | mou2 | zi2 | 禮貌 | lai5 | maau6 |
| 鎚仔 | ceoi4 | zai2 | 隨時 | ceoi4 | si4 | 取消 | ceoi2 | siu1 | 牌照 | paai4 | ziu3 |
| 工人 | gung1 | jan4 | 供應 | gung1 | jing3 | 共享 | gung6 | hoeng2 | 先進 | sin1 | zeon3 |
| 火柴 | fo2 | caai4 | 夥伴 | fo2 | bun6 | 科學 | fo1 | hok6 | 表示 | biu2 | si6 |
| 鉛筆 | jyun4 | bat1 | 圓形 | jyun4 | jing4 | 願望 | jyun6 | mong6 | 降伏 | hong4 | fuk6 |
| 針線 | zam1 | sin3 | 斟水 | zam1 | seoi2 | 浸淫 | zam3 | jam4 | 宣佈 | syun1 | bou3 |
| 藥物 | joek6 | mat6 | 弱點 | joek6 | dim2 | 約束 | joek6 | cuk1 | 絕版 | zyut6 | baan2 |
| 較剪 | gaau3 | zin2 | 教師 | gaau3 | si1 | 交流 | gaau1 | lau4 | 到達 | dou3 | daat6 |
| 油站 | jau4 | zaam6 | 柔軟 | jau4 | jyun5 | 友好 | jau5 | hou2 | 培養 | pui4 | joeng5 |
| 墳墓 | fan4 | mou6 | 焚燒 | fan4 | siu1 | 份量 | fan5 | loeng6 | 零碎 | ling4 | seoi3 |
| 電筒 | din6 | tung2 | 殿堂 | din6 | tong4 | 典禮 | din2 | lai3 | 立法 | lap6 | fat3 |
| 洋蔥 | joeng4 | cung1 | 楊柳 | joeng4 | lau5 | 讓座 | joeng6 | zo6 | 衡量 | hang4 | loeng6 |
