# Supplementary material for: Tonal and syllabic encoding in overt Cantonese Chinese speech production: An ERP study
Source: PLoS One. 2023 Dec 15;18(12):e0295240. doi: 10.1371/journal.pone.0295240 (PMC10723706; doi:10.1371/journal.pone.0295240)
Supplement: Attachment — Submitted filename: comments.docx [file pone.0295240.s002.docx]

This interesting paper tackles the less-studied question: the time course of the lexical tones in the Chinese language and the whole study makes contributions to the field of Chinese word production. The overall design is valid and the results are clearly presented. But I have some questions about the paper.

1. In the last paragraph of the Introduction, the authors mentioned that "if tone and atonal syllable are processed together and affecting the same processing stage, the effects of of tone and atonal syllable relatedness on the ERPs should be non-additive". The logic underlying this claim is not very clear. It will be better for the authors to elaborate on this. Moreover, it will be also better to discuss how the current EEG results relate to these hypotheses again in the discussion section.
2. The authors mentioned Alderete et al. (2019), who also proposed a new model for the tonal encoding in Chinese production. It will be more insightful for the authors to also review their model in this paper and make some comparisions with the previous models proposed by O' Seaghdha et al. (2010) and Roelofs (2015) in the literature review. It will be also better to discuss how the current ERP results could disentangle these different models in the discussion session.
3. The authors mentioned one participant was left-handed. Did this author include this participant in the EEG data analysis? Have you checked whether this participant influenced the overall result?
4. For the distractors, the authors used disyllabic items. Why did the authors not use monosyllabic distractors? Any considerations for this? Have the authors checked that the second syllable of the distractors was not phonologically related to the paired target item?
5. The authors mentioned that the three types of distractors were matched in the syllable frequency and number of homophone. Did the authors match these two variables for both syllables of the distractors?
6. It will be better to provide some details about the preparation of the auditory distractors (were the items pronounced by female or male speakers? What about the average duration of the audios (Was it matched between the three types of distractors)?
7. Have the authors also checked that the second syllable of the Cantonese target items were not phonologically related to the first syllable of the items, as the distractors could also impact the phonological encoding of the second syllable of the items and influenced the ERP results? It will be better for the authors to provide a full list of the stimuli in the appendix.
8. Inspection of the stimulus-locked and response-locked ERPs showed some inconsistencies. In principle, there should be a temporal overlap between the later portion of the stimulus-locked ERP and the early portion of response-locked ERP, which typically would show some converging patterns. However, the later portion of the stimulus-locked ERP showed opposite ERP patterns to those in the early portion of the response-locked ERP. For example, Figure 2A showed that the tonal syllable related condition showed more negativity in the left anterior region than the unrelated condition in the 400-500ms post stimulus onset, but Figure 3A showed the opposite polarity for the tonal syllable related condition in the left anterior region in the -500- -300ms before the speech onset. I noticed the authors had different numbers of participants and different trials for the stimulus-locked and the response-locked ERPs, as well as using different baselines for correction. I think that the authors may need to follow Jeong et al. (2021) and use the same trials and participants and the same baseline for both stimulus-locked and response-locked ERP analysis to see whether such divergent patterns in the paper still existed.

Jeong, H., van den Hoven, E., Madec, S., & Bürki, A. (2021). Behavioral and Brain Responses Highlight the Role of Usage in the Preparation of Multiword Utterances for Production. Journal of Cognitive Neuroscience, 1–34. <https://doi.org/10.1162/jocn_a_01757>

9. The authors mentioned that there are six blocks with each block containing the six distractor conditions. Did the authors counterbalance or randomize the order of the blocks across participants?
10. It seemed that the authors used the voice key automatic detection for the response latencies measurement. Have the authors checked the reliability and accuracy for the detected response latencies using the recorded audios?
11. In the behavioral analysis, the authors excluded the reaction times shorter and longer than 3 SDs, but in the EEG analysis, the authors excluded those trials less than 500 ms and longer than 1500 ms. Why did the authors choose to use different outlier exclusion threshold for the behavioral and EEG data analysis respectively？
12. For the last two paragraphs of 3.2.2, the authors need to provide additional tables to show the results of the further t-test analyses for the Relatedness x Type x Laterality in the -300 to -200ms and the Relatedness x Anteriority x Laterality in the -500 to -400 ms.
13. It seemed that the authors observed a larger priming effect for the tonal syllable related distractors than the atonal syllable related distractors in the behavioral data, but it is unclear how this relates to the EEG results. It seemed that the authors did not observe any amplitude difference for the two types of distractors but only found that the two types of distractors showed distinct patterns in the -400 to -200 and -100 to 0 time window. How could we explain the relatedness between the behavioral data pattern and these EEG results? Why were there two separate time windows showing the different effects? What does this indicate? Some tentative discussion on this would be helpful.
14. Table 3 The authors need to add the table header in the first row to denote the meaning of the numbers.
15. The ERP figures are too blurry with low resolution. Clearer figures need to be provided.
16. other minor issues
    (a) Para. 2, Page 9 (Introduction section) "in a slips of the tongue study" -> "in a study on slips of the tongue"
    (b) Para 2, Page 13 (last paragraph in the introduction) Line 5 "Tonal related condition" -> "Tonal syllable related condition" (Please check the whole paper for consistency)
    (c) Page 15 (2.2 section) "incorrect response (1.6%)" and Page 16 (3.1) "Incorrect (1.58%)" Please keep the consistency of the number of digits.
