# Supplementary material for: Tonal and syllabic encoding in overt Cantonese Chinese speech production: An ERP study
Source: PLoS One. 2023 Dec 15;18(12):e0295240. doi: 10.1371/journal.pone.0295240 (PMC10723706; doi:10.1371/journal.pone.0295240)
Supplement: Attachment — Submitted filename: Response to Reviewers_20231004.docx [file pone.0295240.s003.docx]

Re: Revised version of the manuscript titled “Tonal and syllabic encoding in overt Cantonese Chinese speech production: An ERP study”

We are deeply grateful for the valuable and constructive comments from the reviewers in the previous round of review, based on which substantial revisions to the manuscript have been made. Details of our response to the reviewers’ comments are listed in the following.

Reviewers' comments:

Reviewer #1: This study investigated how syllables and lexical tones are encoded in Cantonese speech production using a picture-word interference task with concurrently recoding electrophysiological signals. Findings suggest that in Cantonese speech production, the atonal syllable of the target is retrieved and then associated with the lexical tone. The manuscript is well written. However, I have some major concerns.

**Response:** We thank Reviewer 1 for the positive evaluations of this work.

1. In the introduction, the authors cited the additive factors method proposed by Sternberg (1969), however, they did not manipulate factors of tone and segments with a factorial design. I think it is questionable to apply the additive factors method in the present design. On the other hand, this logic and paradigm have been widely questioned by many researchers (Koch, Poljac, Müller, & Kiesel, 2018). Therefore, it should be discussed and justified whether the logic is usable in the present study.

**Response:** Thank you for pointing this out. We agree that the present design does not entirely follow a 2 x 2 factorial design and hence it might be questionable if the additive factors logic can readily be applied to this case. Instead of using an independent set of distractor materials to be the baseline (i.e., phonologically unrelated condition) for comparison to fit into and complete the 2 x 2 design, the same three sets of phonologically related distractors (i.e., Tonal Syllable related, Atonal Syllable related, and Tone alone related distractors) were used as the baselines by re-grouping the target-distractor pairs. This way, the same set of picture and distractor materials was used in each phonological condition, and the only difference between related and unrelated conditions was the target-distractor relationship. A within-subjects and within-items design was then achieved. We felt this could best minimize any potential confound associated with the items’ properties, and hence the unrelated baseline conditions were constructed in such a manner. Nevertheless, we agree with Reviewer 1 and have revised the part mentioning about Sternberg’s (1969) additive factors logic. In the revised version, the present design is now motivated by the different views (e.g., early or late tonal encoding) in relation to tonal and syllable encoding as assumed by the different theories of Chinese speech production. Corresponding revisions have been made to the Introduction of the revised version.

2. The major argument is based on the null effect of tonal alone related condition. Please include details of a priori power analysis, a sensitivity analysis, or some other way to make a case that your experiments are sufficiently sensitive or precise enough for your goals.

**Response:** The sample size of the present study is comparable to the previous related ERP (e.g., Qu et al., 2012, 2020) and behavioural (Wang et al., 2019) studies where a significant effect of a single phoneme on Chinese speech planning has been reported. We therefore believe that the current sample size should be sufficient enough to detect the effect of tone alone, if any. Furthermore, additional analyses using the Bayesian approach have been conducted and reported in the revised version. The results of Bayesian analyses showed evidence in support of the H_0_ (i.e., evidence supporting the absence of an effect) in the Tone alone related condition in almost all ERP time windows (with the only one exception showing an inconclusive evidence supporting H_1_). Consequently, regarding the Tone alone related condition, the present ERP data (using the PWI task) suggest that there *is* evidence in favor of the null effect of tone alone instead of an absence of evidence for any effect.

3. I suggest to report Bayesian factor analyses for all statistical results.

**Response:** We thank the Reviewer for this valuable suggestion. It has been done accordingly. The additional results are largely consistent with the results originally reported. The overall picture has become even more clear with the inclusion of Bayesian factor analyses in the revised manuscript.

4. For spoken word production, how to align the time course of processing found in stimulus locked and response locked analyses?

**Response:** We thank the Reviewer for pointing this out. This is a very sensible question. Yet, we are inclined to believe that the present data do not provide us with enough grounds to fully address this concern, future studies (especially studies on the methodological issues related to stimulus-locked and response-locked analyses in particular) might be warrant. As argued by Riès et al. (2013), early stimulus-evoked activities are steeper in their slopes and more transient (as they are well averaged to the stimulus) than the later decision/ selection-evoked activities, it might still be an open issue on how to map the absolute timing between the later part of stimulus-locked analysis and the earlier part of response-locked analysis. Nevertheless, we have adopted the general principles from the previous studies (e.g., Jeong et al., 2019; Laganaro, 2014; Riès et al., 2013) in that stimulus-locked and response-locked analyses are sensitive to the early and late stages of processing respectively. As discussed in the revised manuscript, the effect of atonal syllable priming has been used as a reference to infer the time of when phonological encoding begins (since the bulk of evidence in the literature suggests that the atonal syllable is the first retrievable phonological unit in Chinese phonological planning). With this reference, the relative time course of tonal encoding (e.g., the joint effect of tonal and syllabic priming) was possibly examined in this study.

5. It is difficult to understand why the contrasting findings between tone and segmental units reflect that tone is represented of processed differently from segments. I suggest discuss what are the differences between them.

**Response:** Revised accordingly. Elaborations on the potential differences between tonal and segmental encoding (in the way how they are processed), as postulated by different theories of Chinese speech production, have been included in the revised manuscript.

Reviewer #2: This interesting paper tackles the less-studied question: the time course of the lexical tones in the Chinese language and the whole study makes contributions to the field of Chinese word production. The overall design is valid and the results are clearly presented. But I have some questions about the paper. Please see the attached comments.

**Response:** We thank Reviewer 2 for the positive evaluations of this work.

1.	In the last paragraph of the Introduction, the authors mentioned that "if tone and atonal syllable are processed together and affecting the same processing stage, the effects of of tone and atonal syllable relatedness on the ERPs should be non-additive". The logic underlying this claim is not very clear. It will be better for the authors to elaborate on this. Moreover, it will be also better to discuss how the current EEG results relate to these hypotheses again in the discussion section.

**Response:** Thank you for pointing this out. We agree that the present design does not entirely follow a 2 x 2 factorial design and hence it might be questionable if the additive factors logic can readily be applied to this work. The corresponding discussions have been removed in the revised manuscript. In the revised Introduction, the present study is motivated by the different views (e.g., early or late tonal encoding) in relation to tonal and syllable encoding as assumed by the different theories of Chinese speech production. In addition, elaborated discussions have been included in the Discussion part to relate the present results to the different hypotheses mentioned in Introduction.

2.	The authors mentioned Alderete et al. (2019), who also proposed a new model for the tonal encoding in Chinese production. It will be more insightful for the authors to also review their model in this paper and make some comparisions with the previous models proposed by O' Seaghdha et al. (2010) and Roelofs (2015) in the literature review. It will be also better to discuss how the current ERP results could disentangle these different models in the discussion session.

**Response:** Revised accordingly. Further reviews on Alderete and colleagues’ (2019) model and comparisons between this and other models of Chinese speech production have been provided in the revised manuscript. In addition, elaborated discussions have been added to the Discussion section to discuss how the present ERP data can possibly differentiate the various hypotheses.

3.	The authors mentioned one participant was left-handed. Did this author include this participant in the EEG data analysis? Have you checked whether this participant influenced the overall result?

**Response:** The left-handed participant was included in the stimulus-locked analysis but was not in the response-locked analysis. Nevertheless, a similar pattern of result was observed whether or not this participant was included in the ERP data analysis, the conclusions reached in this study would not be affected by this single case.

4.	For the distractors, the authors used disyllabic items. Why did the authors not use monosyllabic distractors? Any considerations for this? Have the authors checked that the second syllable of the distractors was not phonologically related to the paired target item?

**Response:** Thank you for this suggestion. We had considered this possibility before. It was only that if monosyllabic distractors were used, the Tonal Syllable related distractors will then become identical (not merely homophonous) to the first syllable/character/morpheme of the target. The target and tonal syllable related distractors will then share many aspects of similarity (e.g., semantic, morphological, phonological, and orthographic) apart from the phonological one. The situation would be different if disyllabic distractors were used, where the second syllable of the distractor could provide the necessary context guiding the interpretation of the first syllable (which was only homophonous but not identical to the target). Disyllabic distractors were therefore adopted in the present study. Cautions have been taken to ensure that the second syllable of the distractor did not share the same atonal syllable with the targets.

5.	The authors mentioned that the three types of distractors were matched in the syllable frequency and number of homophone. Did the authors match these two variables for both syllables of the distractors?

**Response:** Thank you for pointing this out. Clarifications have been made in the revised manuscript (section 2.2 Materials and apparatus).

6.	It will be better to provide some details about the preparation of the auditory distractors (were the items pronounced by female or male speakers? What about the average duration of the audios (Was it matched between the three types of distractors)?

**Response:** Provided accordingly. Please see the revised section 2.2. The mean duration of the auditory distractors was matched across the three distractor type conditions.

7.	Have the authors also checked that the second syllable of the Cantonese target items were not phonologically related to the first syllable of the items, as the distractors could also impact the phonological encoding of the second syllable of the items and influenced the ERP results? It will be better for the authors to provide a full list of the stimuli in the appendix.

**Response:** Cautions have been taken to ensure that the two syllables of the distractor did not overlap in their atonal syllable. A full list of the stimuli is included in the Appendix of the revised manuscript.

8.	Inspection of the stimulus-locked and response-locked ERPs showed some inconsistencies. In principle, there should be a temporal overlap between the later portion of the stimulus-locked ERP and the early portion of response-locked ERP, which typically would show some converging patterns. However, the later portion of the stimulus-locked ERP showed opposite ERP patterns to those in the early portion of the response-locked ERP. For example, Figure 2A showed that the tonal syllable related condition showed more negativity in the left anterior region than the unrelated condition in the 400-500ms post stimulus onset, but Figure 3A showed the opposite polarity for the tonal syllable related condition in the left anterior region in the -500- -300ms before the speech onset. I noticed the authors had different numbers of participants and different trials for the stimulus-locked and the response-locked ERPs, as well as using different baselines for correction. I think that the authors may need to follow Jeong et al. (2021) and use the same trials and participants and the same baseline for both stimulus-locked and response-locked ERP analysis to see whether such divergent patterns in the paper still existed.

Jeong, H., van den Hoven, E., Madec, S., & Bürki, A. (2021). Behavioral and Brain Responses Highlight the Role of Usage in the Preparation of Multiword Utterances for Production. Journal of Cognitive Neuroscience, 1–34. https://doi.org/10.1162/jocn_a_01757

**Response:** We thank Reviewer 2 for this valuable comment. We agree that the different polarities of the effects mentioned above might be related to the differences between stimulus-locked and response-locked analyses presently conducted. Yet, we also noticed that it might not be very uncommon for researchers to adopt different baselines for stimulus-locked and response-locked analyses (e.g., Riès et al., 2013). In our response-locked analysis, the 200-ms period between 2000 ms and 1800 ms pre-response onset was taken as the baseline, to make sure that the baseline period would fall entirely within the time of when the fixation point was presented which was the same for all trials and all conditions included in the analysis. Another merit of this approach is that the time lag between the baseline period and the time window of interest (i.e., the 500-ms pre-response onset) could be kept constant across different conditions and trials. This time lag, however, would be varied across conditions (and trials) if a fixed baseline period (say for instance, the 200-ms pre-stimulus onset) was chosen for the response-locked analysis (since the response onset varies across conditions and trials).

Nevertheless, we have adopted the general principles from the previous related studies (e.g., Jeong et al., 2019; Laganaro, 2014; Riès et al., 2013) in that stimulus-locked and response-locked analyses are sensitive to the early and late stages of processing respectively. As discussed in the revised manuscript, the effect of atonal syllable priming has been used as a reference to infer the time of when phonological encoding begins (since the bulk of evidence in the literature suggests that the atonal syllable is the first retrievable phonological unit in Chinese phonological planning). With this reference, the relative time course of tonal encoding (e.g., the joint effect of tonal and syllabic priming) was possibly examined in this study. Although it might still be an open issue regarding the different polarities of the effects mentioned above, the current ERP results are clear enough to inform the various views on the *relative* time course of tonal (early or late encoding) and syllabic encoding in Chinese speech production.

9.	The authors mentioned that there are six blocks with each block containing the six distractor conditions. Did the authors counterbalance or randomize the order of the blocks across participants?

**Response:** Clarifications have been provided accordingly (please see revised section 2.3 Design and procedure).

10.	It seemed that the authors used the voice key automatic detection for the response latencies measurement. Have the authors checked the reliability and accuracy for the detected response latencies using the recorded audios?

**Response:** We thank Reviewer 2 for this valuable suggestion. This was not included in our original research plan and proposal so no audio recording of the participants’ responses was taken for this purpose. This is a limitation of the present study. We totally agree that it would be very useful to have this included and will take this into consideration in our future studies.

11.	In the behavioral analysis, the authors excluded the reaction times shorter and longer than 3 SDs, but in the EEG analysis, the authors excluded those trials less than 500 ms and longer than 1500 ms. Why did the authors choose to use different outlier exclusion threshold for the behavioral and EEG data analysis respectively？

**Response:** Excluding behavioural trials with reaction times beyond 3 SDs was a practice generally used in the past behavioural studies. In our ERP analysis, trials with RT less than 500 ms or longer than 1500 ms were discarded. Trials with RT less than 500 ms were excluded so as to make sure that the response onset would not occur within the first 500 ms post-stimulus onset where the stimulus-locked analysis was conducted, to minimize the impacts of motor related artefacts. Trials with RT longer than 1500 ms were also excluded because for those trials, the baseline period (i.e., the 2000 ms to 1800 ms pre-response onset) would fall out of the period of when the fixation point was presented on the screen. Nevertheless, the difference in exclusion criterion was due mainly to practical methodological reasons, and there was a considerable degree of overlap between these different criterion (i.e., trials with RT beyond 3 SDs were also the trials with RT lower than 500 ms or longer than 1500 ms).

12.	For the last two paragraphs of 3.2.2, the authors need to provide additional tables to show the results of the further t-test analyses for the Relatedness x Type x Laterality in the -300 to -200ms and the Relatedness x Anteriority x Laterality in the -500 to -400 ms.

**Response:** Provided accordingly.

13.	It seemed that the authors observed a larger priming effect for the tonal syllable related distractors than the atonal syllable related distractors in the behavioral data, but it is unclear how this relates to the EEG results. It seemed that the authors did not observe any amplitude difference for the two types of distractors but only found that the two types of distractors showed distinct patterns in the -400 to -200 and -100 to 0 time window. How could we explain the relatedness between the behavioral data pattern and these EEG results? Why were there two separate time windows showing the different effects? What does this indicate? Some tentative discussion on this would be helpful.

**Response:** We thank Reviewer 2 for this valuable comment. Additional correlation analyses have been conducted and reported in the revised manuscript to provide supplemental evidence to show the relationship between the observed behavioural and ERP effects. In addition, further elaborated discussions, as well as a tentative account, for the finding that two separate time windows in response-locked analysis showing the different effects of the Tonal Syllable and Atonal Syllable conditions are included in the revised Discussion section.

14.	Table 3 The authors need to add the table header in the first row to denote the meaning of the numbers.

**Response:** Revised accordingly.

15.	The ERP figures are too blurry with low resolution. Clearer figures need to be provided.

**Response:** Revised accordingly.

16.	other minor issues

(a)	Para. 2, Page 9 (Introduction section) "in a slips of the tongue study" -> "in a study on slips of the tongue"

**Response:** Revised accordingly.

(b)	Para 2, Page 13 (last paragraph in the introduction) Line 5 "Tonal related condition" -> "Tonal syllable related condition" (Please check the whole paper for consistency)

**Response:** Revised accordingly.

(c)	Page 15 (2.2 section) "incorrect response (1.6%)" and Page 16 (3.1) "Incorrect (1.58%)" Please keep the consistency of the number of digits.

**Response:** Revised accordingly.
