# Supplementary material for: Tonal and syllabic encoding in overt Cantonese Chinese speech production: An ERP study
Source: PLoS One. 2023 Dec 15;18(12):e0295240. doi: 10.1371/journal.pone.0295240 (PMC10723706; doi:10.1371/journal.pone.0295240)
Supplement: Attachment — Submitted filename: response letter 20231109.docx [file pone.0295240.s004.docx]

**Re: 2^nd^ revision of the manuscript titled “Tonal and syllabic encoding in overt Cantonese Chinese speech production: An ERP study”**

We are deeply grateful for the valuable and constructive comments from the reviewers in the last two rounds of review, based on which revisions to the manuscript have been made. Details of our response to the reviewers’ comments are listed in the following.

Reviewers' comments:
**Reviewer #1:** Authors revised the manuscript according to my reviews. I think it is acceptable for publication in PLoS ONE.

**Response:**

We thank Reviewer 1 for the positive evaluation of this work.

**Reviewer #2:** The questions raised have been mostly addressed. However, there are still two issues for the authors.

First, the use of different baseline for stimulus-locked and response-locked ERP was justified by the authors' explanations. However, it will be better for the authors to check if the same participants and the same trials used for stimulus-locked and response-locked ERPs produced the similar results as reported in the current version, as this could ensure that the opposite polarity between the later portion of stimulus-locked ERP and the early portion of response-locked ERP was not just due to difference in participants and trials (given the intersubject and intertrial variability typically observed in ERP data).

**Response:**

We thank Reviewer 2 for the suggestion. We agree with Reviewer 2 that multiple factors may contribute to the different ERP waveform patterns between the end of the stimulus-locked time window and the beginning of the response-locked time window. To examine the potential influence of inter-subject variability, additional ERP analyses have been conducted with the data from the 24 participants which were previously included in *both* stimulus-locked and response-locked analyses. This way, the same group of participants (though with a reduced sample size) was included in the additional analysis for both stimulus-locked and response-locked analyses (using similar procedures as previously described). The results of the additional analyses were largely similar to the stimulus-locked and response-locked analysis results previously presented in the report (details are shown in the tables below). Specifically, significant ERP effects (i.e., significant differences between related and unrelated conditions in mean ERP amplitudes) were observed in both Tonal Syllable and Atonal Syllable distractor conditions in the time window of 400 to 500 ms post-stimulus onset (i.e., the end of the stimulus-locked time window), and the effects were confined to the anterior ROIs. Significant ERP effects were also observed in the Tonal Syllable and Atonal Syllable conditions in the response-locked analysis across various time windows, including the -500 to -400 ms pre-response onset (i.e., the beginning of the response-locked time window). Importantly, the polarity of these two ERP effects (i.e., 400 to 500 ms post-stimulus onset and -500 to -400 ms pre-response onset) were reversed (as evident by the signs of the *t* values shown in the corresponding tables below) which was the same as the waveform patterns previously observed. These additional results suggest that the variation in participant numbers (e.g., N = 28 in stimulus-locked analysis vs. N = 26 in response-locked analysis) might probably not be a crucial factor contributing to the observed contrastive waveform patterns.

| Additional analysis 1 (N = 24) | | | |  | |  | |  | |  | | |  |  |  |
| --- | --- | --- | --- | --- | --- | --- | --- | --- | --- | --- | --- | --- | --- | --- | --- |
| Effects of relatedness at different levels of laterality across different distractor conditions during the time windows of 300 – 400 ms and 400 – 500 ms post-stimulus onset | | | | | | | | | | | | | | | |
|  |  | | |  | |  | |  | |  | | |  |  |  |
|  | | | | | | Laterality | | | | | | | | | |
|  |  |  |  |  |  | Left | | | | Middle | | | | Right | |
| Time window | Distractor Type | | | *df* | | *t* | | *p* | | *t* | | | *p* | *t* | *p* |
| 300 – 400 ms | Tonal Syllable | | | 23 | | -1.753 | | 0.093 | | 1.367 | | | 0.185 | 0.046 | 0.964 |
|  | Atonal Syllable | | | 23 | | -2.262 | | 0.033 | | 2.624 | | | 0.015 | -0.034 | 0.973 |
|  | Tone alone | | | 23 | | -0.207 | | 0.838 | | -0.489 | | | 0.63 | -0.363 | 0.72 |
| 400 – 500 ms | Tonal Syllable | | | 23 | | -3.102 | | 0.005* | | 2.927 | | | 0.008 | 0.456 | 0.653 |
|  | Atonal Syllable | | | 23 | | -3.265 | | 0.003* | | 1.906 | | | 0.069 | 1.094 | 0.285 |
|  | Tone alone | | | 23 | | 0.788 | | 0.439 | | -0.871 | | | 0.393 | 0.21 | 0.836 |
| * Significant at alpha = .0055 level | | | | | |  | |  | |  | | |  |  |  |
| Additional analysis 2 (N = 24) | | |  | |  | |  | |  | |  |  |  |  |  |
| Effects of relatedness at different levels of anteriority across different distractor conditions and time windows during the 500-ms pre-response onset | | | | | | | | | | | |  |  |  |  |
|  | |  |  | |  | |  | |  | |  |  |  |  |  |
|  | |  |  | | Anteriority | | | | | | |  |  |  |  |
|  | |  |  | | Anterior | | | | Posterior | | |  |  |  |  |
| Time window | | Distractor Type | *df* | | *t* | | *p* | | *t* | | *p* |  |  |  |  |
| -500 to -400 ms | | Tonal Syllable | 23 | | 5.582 | | < .001* | | -5.218 | | < .001* |  |  |  |  |
|  | | Atonal Syllable | 23 | | 4.281 | | < .001* | | -4.039 | | < .001* |  |  |  |  |
|  | | Tone alone | 23 | | 0.591 | | 0.56 | | 0.016 | | 0.987 |  |  |  |  |
| -400 to -300 ms | | Tonal Syllable | 23 | | 3.562 | | 0.002* | | -3.36 | | 0.003* |  |  |  |  |
|  | | Atonal Syllable | 23 | | 1.614 | | 0.12 | | -1.143 | | 0.265 |  |  |  |  |
|  | | Tone alone | 23 | | -0.395 | | 0.696 | | 0.668 | | 0.511 |  |  |  |  |
| -300 to -200 ms | | Tonal Syllable | 23 | | 0.466 | | 0.646 | | -0.266 | | 0.792 |  |  |  |  |
|  | | Atonal Syllable | 23 | | -0.952 | | 0.351 | | 1.188 | | 0.247 |  |  |  |  |
|  | | Tone alone | 23 | | 1.479 | | 0.153 | | -1.601 | | 0.123 |  |  |  |  |
| -200 to -100 ms | | Tonal Syllable | 23 | | -4.867 | | < .001* | | 4.352 | | < .001* |  |  |  |  |
|  | | Atonal Syllable | 23 | | -3.905 | | < .001* | | 3.35 | | 0.003* |  |  |  |  |
|  | | Tone alone | 23 | | 0.126 | | 0.901 | | -1.084 | | 0.289 |  |  |  |  |
| -100 to 0 ms | | Tonal Syllable | 23 | | -5.943 | | < .001* | | 5.642 | | < .001* |  |  |  |  |
|  | | Atonal Syllable | 23 | | -1.765 | | 0.091 | | 1.004 | | 0.326 |  |  |  |  |
|  | | Tone alone | 23 | | -1.234 | | 0.23 | | 1.194 | | 0.244 |  |  |  |  |
| * Significant at alpha = .0083 level | | |  | |  | |  | |  | |  |  |  |  |  |

| Additional analysis 3 (N = 24) | |  |  |  |  |  |  |  |
| --- | --- | --- | --- | --- | --- | --- | --- | --- |
| Effects of relatedness at different levels of laterality across different distractor conditions during the time window of -300 to -200 ms pre-response onset | | | | | | | | |
|  |  |  |  |  |  |  |  |  |
|  | | | Laterality | | | | | |
|  |  |  | Left | | Middle | | Right | |
| Time window | Distractor Type | *df* | *t* | *p* | *t* | *p* | *t* | *p* |
| -300 to -200 ms | Tonal Syllable | 23 | 0.209 | 0.836 | -0.874 | 0.391 | 1.581 | 0.128 |
|  | Atonal Syllable | 23 | -3.702 | 0.001* | 2.267 | 0.033 | 2.163 | 0.041 |
|  | Tone alone | 23 | 1.317 | 0.201 | -2.017 | 0.055 | 0.549 | 0.589 |
| * Significant at alpha = .0055 level | |  |  |  |  |  |  |  |

Nevertheless, a further screening at the trial level by retaining only those trials that concurrently fulfilled the inclusion criterion (e.g., < ±120µV) of *both* stimulus-locked and response-locked analyses has not been performed. We refrained from performing this as to prevent the issue of double screening which may further reduce the data size and unavoidably limit our analyses to a restricted set of data sample. We do admit that it might be a limitation which requires further study. Future research is needed to further examine the different approaches to performing stimulus-locked and response-locked EEG analyses. Nevertheless, as the focus of this study is the relatively time course of syllabic and tonal encoding in Cantonese spoken word production, the results currently reported should be clear enough to inform this target issue.

**Reviewer #2 (Cont.):**

Second, the stimulus selection/control in the current experiment was actually not very perfect. I noticed that some of the target items contained two different syllables with the same tone (e.g., fei1 kei1, 'plane'). Since the tone is the focus of the current study, it will be better to try to avoid such items in order to obtain a more reliable effect, as it is unclear whether the tone itself could potentially show some effect in ERP. Even though previous studies did not report the tonal overlap alone could produce priming effect in behavioural data (in Cantonese and Mandarin), there is some evidence that the tonal overlap alone may potentially cause an interference effect in Mandarin (see Chen et al., 2002). Moreover, the rime sharing (e.g., fei1 kei1, 'plane') may also introduce some unknown influence to the processing.

**Response:**

Once again, we thank Reviewer 2 for this valuable comment. It is indeed the case that for some of the targets, their two syllables share the same tone (or having a similar sub-syllabic component). This is also a constraint that we faced when constructing the materials for this study. Given there are only six lexical tones in Cantonese, it is not easy (if not impossible) to find good enough of disyllabic targets, composed by two syllables having different tones, which are familiar to the participants (based on the rating results) and can easily be picturized and named. In addition, it also requires to have a matched atonal syllable available (a similar syllable but with a different tone) to form the atonal syllable distractor condition. Given the bulk of evidence in the literature suggests that the atonal syllable is the primary planning unit in Cantonese and Mandarin Chinese speech production, we therefore matched the two syllables of each of our disyllabic targets at the atonal syllable level (i.e., each target must be composed by two different atonal syllables). Furthermore, as a within-items design was adopted in this study, that is, the same set of targets were used across different distractor type conditions. So, if there were indeed effects from “within” the targets, such effects should be constant across different conditions. However, one may concern if these potential within-target effects would interact with the priming effects in different ways across different conditions. This is an empirical question requires further investigation and we admit that further research is needed to examine this possibility. We thank Reviewer 2 for this comment. A footnote has therefore been added in the revised manuscript in response to this comment.
